# Association of hemoglobin variation and hospital mortality in patients with traumatic brain injury at high altitude

**DOI:** 10.3389/fneur.2025.1669136

**Published:** 2025-09-26

**Authors:** Zeqing Wang, Zekui Zhang, Zhenjun Liu

**Affiliations:** ^1^Department of Critical Medicine, People's Hospital of Aba Tibetan and Qiang Autonomous Prefecture of Sichuan Province, Maerkang, China; ^2^Department of Critical Medicine, Sichuan Clinical Research Center for Cancer, Sichuan Cancer Hospital and Institute, Sichuan Cancer Center, University of Electronic Science and Technology of China, Chengdu, China

**Keywords:** traumatic brain injury, hemoglobin variation, mortality, hemoglobin, Glasgow coma scale (GCS)

## Abstract

**Backgroud:**

Traumatic brain injury (TBI) ranks among the leading causes of death worldwide. However, the association between hemoglobin variation (ΔHb) and hospital mortality in TBI patients at high altitude remains uninvestigated.

**Method:**

This retrospective cohort study was conducted from January 2020 to March 2025 in the Tibetan Plateau region, enrolling 191 patients who resided at an average altitude of 3,000 m. ΔHb (peak Hb-nadir Hb) during the hospitalization, related covariates and hospital mortality were collected. Backward stepwise multivariable logistic regression was used to select key variables. The non-linear relationship between ΔHb and mortality was investigated using the multivariable fractional polynomial (MFP) method. The threshold effects of ΔHb were explored through two-piecewise logistic regression models.

**Results:**

Logistic regression showed that ΔHb was independently and significantly associated with hospital mortality (OR = 1.08, 95% CI: 1.02–1.15, *P* = 0.005) after adjustment for nadir Hb, diffuse axonal injury and GCS (Glasgow coma scale) score. A cubic non-linear relationship between ΔHb and hospital mortality was revealed (*P* for non-linearity = 0.010), with an inflection point at 19.8 g/L. Additionally, an interaction effect between ΔHb and GCS score was found (*P* = 0.035).

**Conclusions:**

In the clinical management of high-altitude TBI patients, our findings suggest that those with a ΔHb > 20 g/L, and a substantially elevated ΔHb and a low GCS score have an increased risk of mortality. A study investigating interventional strategies aimed at reducing ΔHb in TBI patients is warranted.

## Introduction

Traumatic brain injury (TBI) is among the leading global causes of death and long-term disability, affecting 18 to 23 million people each year worldwide (1). TBI classification is multi-dimensional, and is based on severity, pathological features, imaging findings, and functional outcomes. The most common neurological scale used for TBI neurological examinations and TBI prognose determination is the Glasgow Coma Scale (GCS), which assesses the extent of the best motor response, verbal response, and eye opening. Depending on the GCS score, the course of traumatic brain injury can be classified as mild (a GCS score of 13–15), moderate (a GSC score of 9–12), or severe (a GCS score of 3–8) (2). A key focus in TBI management is on preventing and alleviating secondary brain injuries that occur subsequent to initial trauma. These secondary insults, such as hypoxia, hypotension, cerebral edema, and ischemia, can exacerbate the primary brain damage and significantly influence patient outcomes (3).

Hemoglobin (Hb) levels may be influenced by multifactorial causes such as acute blood loss, hemodilution, impaired RBC production, and transfusion-related factors (4, 5). Therefore, Hb dynamics are shaped by TBI severity, pathological processes, and clinical management. The association between Hb levels and outcomes in TBI patients has been extensively investigated, yet remains inconclusive (6, 7). Recently, two landmark randomized controlled trials (8, 9) have shown that maintaining an appropriate Hb level (Hb ≥ 90–100 g/L) may potentially promote neurological outcomes. However, the association between hemoglobin variation (ΔHb) and the outcomes of TBI patients, especially hospital mortality has not been investigated.

The effects of perioperative ΔHb on adverse outcomes in patients after lung resection and major gastrointestinal surgery have been reported (10–12). It has been observed that hemoglobin concentration increases at high altitudes (13), which may lead to a predicted higher ΔHb in TBI patients compared to those residing in plain areas. To the best of our knowledge, the association between ΔHb and hospital mortality in TBI patients at high altitude remains uninvestigated in the existing literature.

## Methods

### Study design and setting

This retrospective cohort study was conducted from January 2020 to March 2025 at the tertiary Aba Prefecture People's Hospital. The study protocol was approved by the hospital's ethical review board (No. 2025-08). Informed consent from the patients or family members was waived due to the retrospective design. Managements for the TBI patients were conducted in accordance with the established guideline (14).

### Participants

Patients with TBI in the Aba Tibetan and Qiang Autonomous Prefecture (average altitude of 3,000 m) were enrolled. The inclusion criteria were as follows: (a) aged 18 years or older; (b) admitted to the ICU (intensive care unit); (c) at least two Hb values available during the hospital stay. TBI patients from non-Aba areas, and with missing data were excluded.

### Data collection

Hb values at admission (baseline Hb), nadir Hb, peak Hb and ΔHb (peak Hb-nadir Hb) during hospitalization were collected. Covariates were selected based on clinical experience and literature review (9, 15). The covariates, including age, gender, GCS (Glasgow coma scale) score, comorbidities and Marshall CT score, were retrieved at hospital admission from the EMR (electronic medical record) system. Other covariates including blood transfusion, trauma type, and neurological surgical interventions, and the outcomes including ICU length of stay, hospital length of stay and hospital mortality, were recorded during the hospitalization.

### Statistical analysis

The statistical analysis was performed using STATA 18.0 (StataCorp, College Station, TX, USA). Following the Shapiro-Wilk normality test, quantitative variables were presented as mean±standard deviation or median with interquartile range (25th to 75th percentile) as appropriate. Categorical variables were expressed as frequencies (percentages). For group comparisons, the Student's *t*-test or Wilcoxon test was used for quantitative variables, and the chi-square test or Fisher's exact test was applied to categorical variables, as suitable.

Backward stepwise multivariable logistic regression was used to select key variables, and the variables with *P* < 0.05 remained in the final regression. The non-linear relationship between ΔHb and mortality was investigated via the multivariable fractional polynomial (MFP) method (16, 17). The threshold effects of ΔHb were assessed via two-piecewise logistic regression models.

Sensitivity analysis was conducted by including patients with missing data, which were addressed using multiple imputation by chained equations (MICE), and the estimated results were combined using Rubin's rule. All *P*-values were calculated as two-sided tests, and *P* < 0.05 was considered statistically significant.

## Results

### Patients characteristics

**Figure 1:**
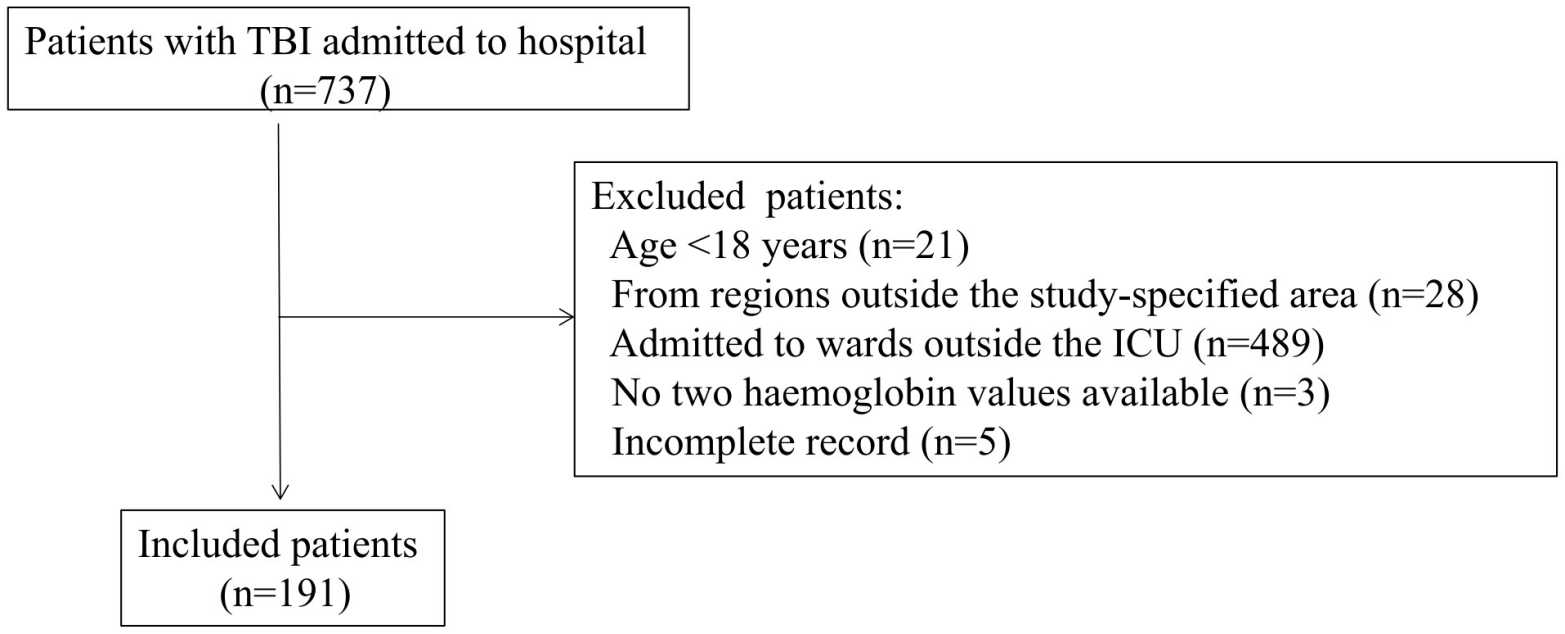
Flowchart of the patients enrollment. ICU, intensive care unit; TBI, traumatic brain injury.

We enrolled 191 patients with TBI during the study period. The enrollment flowchart is shown in Figure 1. The majority of participants were young (47.1 ± 16.0 years), male gender (76.4%) and had severe TBI (58.6%), as shown in Table 1.

**Table 1:** Characteristics of enrolled patients.

**Variables**	**Survivors**	**Non-survivors**	**Total**	***P*-value**
*N*	141 (73.8%)	50 (26.2%)	191 (100.0%)	
Age (years)	47.7 ± 16.9	45.4 ± 13.2	47.1 ± 16.0	0.394
Male sex	106 (75.2%)	40 (80.0%)	146 (76.4%)	0.490
Peak Hb (g/L)	138 (124–161)	134 (110–157)	137 (122–161)	0.175
Nadir Hb (g/L)	107 (90–130)	94 (69–116)	104 (87–127)	0.004
ΔHb (g/L)	31 (23–43)	38 (31–62)	32 (24–47)	0.003
Baseline Hb (g/L)	136 (122–161)	129 (110–156)	135 (119–160)	0.083
Blood transfusion	64 (45.4%)	32 (64.0%)	96 (50.3%)	0.024
**Comorbidity**				
Cardiovascular system diseases	9 (6.4%)	4 (8.0%)	13 (6.8%)	0.746
Endocrine system diseases	5 (3.5%)	1 (2.0%)	6 (3.1%)	1.000
Respiratory system diseases	6 (4.3%)	4 (8.0%)	10 (5.2%)	0.292
Nervous system diseases	2 (1.4%)	1 (2.0%)	3 (1.6%)	1.000
Digestive system diseases	5 (3.5%)	3 (6.0%)	8 (4.2%)	0.433
**GCS**				
13–15	37 (26.2%)	3 (6.0%)	40 (20.9%)	0.002
9–12	31 (22.0%)	8 (16.0%)	39 (20.4%)	
3–8	73 (51.8%)	39 (78.0%)	112 (58.6%)	
**Marshall CT score**				
1–2	68 (48.2%)	16 (32.0%)	84 (44.0%)	0.036
3–4	48 (34.0%)	17 (34.0%)	65 (34.0%)	
5–6	25 (17.7%)	17 (34.0%)	42 (22.0%)	
**Trauma type**				
Cerebral Contusion/ICH	127 (90.1%)	47 (94.0%)	174 (91.1%)	0.566
Subarachnoid hemorrhage	113 (80.1%)	45 (90.0%)	158 (82.7%)	0.131
Subdural hematoma	69 (48.9%)	24 (48.0%)	93 (48.7%)	1.000
Epidural hematoma	50 (35.5%)	17 (34.0%)	67 (35.1%)	0.852
Diffuse axonal injury	26 (18.4%)	26 (52.0%)	52 (27.2%)	<0.001
Skull fracture	113 (80.1%)	42 (84.0%)	155 (81.2%)	0.675
**Operative intervention**				
Hematoma evacuation	31 (22.0%)	9 (18.0%)	40 (20.9%)	0.687
Decompressive craniectomy	17 (12.1%)	8 (16.0%)	25 (13.1%)	0.472
Burr hole drainage	12 (8.5%)	0 (0.0%)	12 (6.3%)	0.038
Cranioplasty	5 (3.5%)	1 (2.0%)	6 (3.1%)	1.000
ICU stay (days)	16 (5–3)	3 (2–8)	9 (3–26)	<0.001
Hospital stay (days)	7 (4–13)	3 (1–8)	6 (3–12)	<0.001

Hb, hemoglobin; GCS, Glasgow coma scale; ICH, intracerebral hemorrhage; ICU, intensive care unit.

There were significant differences between the survivors and non-survivors in terms of TBI severity (GCS score), nadir Hb, Marshall CT score, blood transfusion, diffuse axonal injury, burr hole drainage, ICU length of stay, and hospital length of stay (*P* < 0.05).

ΔHb had a median value of 32 g/L, with a minimum of 2 g/L and a maximum of 125 g/L, and showed a heavily right-tailed skewed distribution (Supplementary Figure S1). Notably, ΔHb significantly differed between survivors and non-survivors [31 (23–43) g/L vs. 38 (31–62) g/L, *P* = 0.003].

### Association between ΔHb and hospital mortality

ΔHb and covariates including age, gender, peak Hb, nadir Hb, baseline Hb, GCS score, comorbidities, Marshall CT score, blood transfusion, trauma type, and neurological surgical interventions were included in the backward logistic regression. The regression identified that ΔHb (OR = 1.08, 95%CI: 1.02–1.15, *P* = 0.005) was independently and significantly associated with hospital mortality after adjustment for nadir Hb (OR = 0.98, 95% CI: 0.97–0.99, *P* = 0.012), diffuse axonal injury (OR = 2.98, 95% CI: 1.36–6.51, *P* = 0.006) and GCS (OR = 0.86, 95% CI: 0.77–0.96, *P* = 0.011), as shown in Table 2.

**Table 2:** The association of ΔHb and mortality.

**Model**	**OR**	**95%CI**	***P*-value**
The multivariable logistic regression model^a^	1.08	1.02–1.15	0.005
**The two-piecewise logistic regression model^b^**			
≤19.8	0.89	0.81–0.98	0.018
> 19.8	1.03	1.01–1.04	0.014
Log-likelihood ratio test			0.002

OR, odds ratio; CI, confidence interval.

^a^Analyzed after adjustment for nadir Hb, diffuse axonal injury and GCS.

^b^Analyzed after adjustment for nadir Hb, diffuse axonal injury and GCS.

### Non-linear relationship and threshold effect analysis of ΔHb

**Figure 2:**
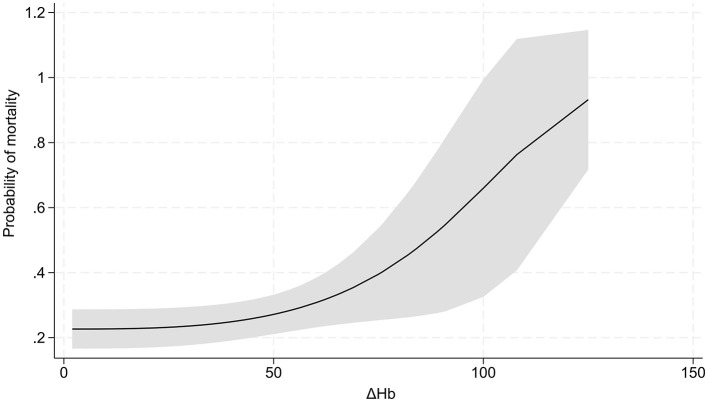
Non-linear association of ΔHb and hospital mortality.

MFP showed a cubic non-linear relationship between ΔHb and hospital mortality (*P* for non-linearity = 0.010, Figure 2). Two-piecewise logistic regression analysis revealed that 19.8 g/L of ΔHb represented an inflection point. Below this infection point, ΔHb was significantly negatively associated with mortality (OR = 0.89, 95% CI:0.81–0.98, *P* = 0.018), whereas beyond this point, ΔHb was positively associated with mortality (OR = 1.03, 95% CI:1.01–1.04, *P* = 0.014). The log-likelihood ratio test supported the significant superiority of the segmented model over the linear model (*P* = 0.002).

### Interaction effect between ΔHb and GCS

**Figure 3:**
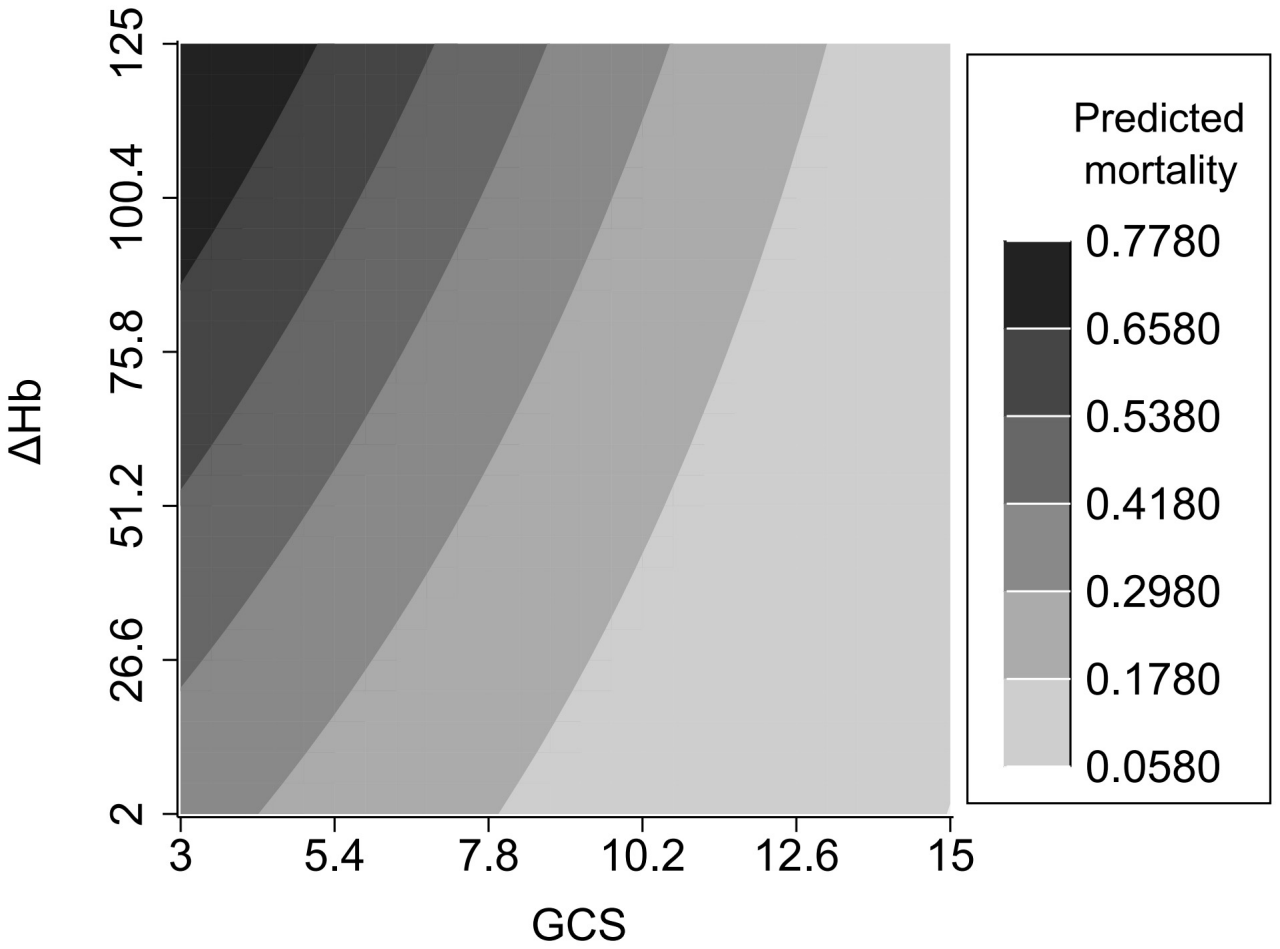
The interaction effect of ΔHb and GCS. GCS, Glasgow coma scale.

**Figure 4:**
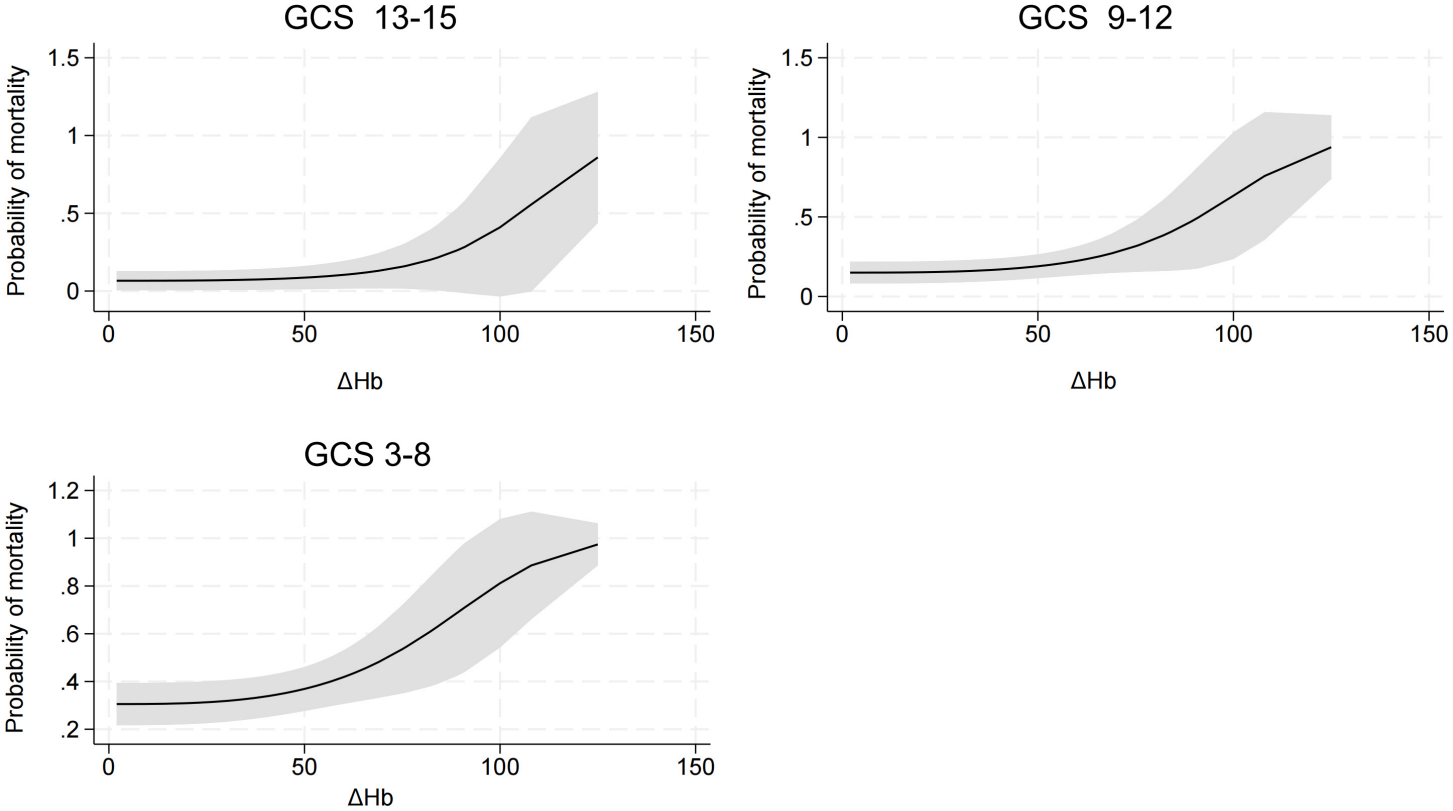
Association of ΔHb and hospital mortality stratified by GCS. GCS, Glasgow coma scale.

An interaction effect between ΔHb and GCS score was found (*P* = 0.035). Compared to TBI patients with lower ΔHb and higher GCS score, those with higher ΔHb and lower GCS score had an increased risk of hospital mortality (Figure 3). In addition, detrimental effect of elevated ΔHb was more considerable in the severe TBI patients than in the moderate and mild patients. Figure 4 demonstrats increased baseline and marginal probabilities for hospital mortality caused by ΔHb stratified by GCS.

### Sensitivity analysis

We enrolled 5 patients with missing data, and a total of 196 patients for analysis after MICE. The association of ΔHb and mortality was significant after adjustment for nadir Hb, diffuse axonal injury and GCS score (OR = 1.07, 95% CI: 1.03–1.13, *P* = 0.006). Furthermore, the non-liearity relationship between ΔHb and mortality (*P* for non-linearity = 0.019), inflection point (19.8 g/L) of ΔHb and interaction effect between ΔHb and GCS were consistent (*P* for interaction = 0.033).

## Discussion

Residents at high altitudes adapt to reduced inspiratory oxygen partial pressure through stimulating erythropoiesis, iron hypermetabolism (18) and adaptive mitochondrial plasticity (19). Given the unique physiological characteristics of Hb at high altitudes, exploring the potential influence of ΔHb on patient prognosis could provide valuable insights for the management and treatment strategies of high-altitude TBI patients. Our study revealed that ΔHb was an independent factor associated with hospital mortality in high-altitude TBI patients after adjustment for nadir Hb and other covariates. In general TBI patients, the linkage between lower Hb levels and mortality has been documented (15, 20). Consistent with these findings, our study also indicated deceased Hb was significantly associated with mortality in TBI patients at high altitude.

In addition, our study revealed the injurious impact of ΔHb on outcomes in TBI patients at high altitude. Besides, we identified a threshold effect of ΔHb on hospital mortality, with 19.8 g/L as an inflection point. Our study unraveled an exponential growth pattern between ΔHb and mortality, which indicated elevated ΔHb led to a considerably increased probability of mortality. Previous studies have demonstrated that elevated ΔHb is a significant factor associated with poor outcomes in patients with aneurysmal subarachnoid hemorrhage (21), renal disease undergoing maintenance hemodialysis (22), myocardial injury (23) and decompensated heart failure (24). These growing body of evidence confirms that hemoglobin fluctuations over time (ΔHb)—rather than static hemoglobin levels—are robust predictors of poor outcomes in various acute and chronic conditions. This aligns with our core finding that ΔHb exerts an unfavarable impact on TBI patients at high altitude. Another key novelty of our study is the exponential relationship between ΔHb and mortality—once ΔHb exceeds inflection point, mortality risk rises exponentially. Aformentioned existing literature reports linear relationships between Hb changes and outcomes, which highlights the unique characteristics of ΔHb's impact in our cohort. This discrepancy may stem from the synergistic stress of high-altitude hypoxic environment and TBI.

In clinical practice, emphasis should be placed on TBI patients with a ΔHb > 19.8 g/L; furthermore, compared with TBI patients with a mild increased ΔHb, those with a significant increased ΔHb have a higher risk of death. Whether interventions aimed at reducing ΔHb can decrease mortality remains to be confirmed by prospective studies.

An interaction effect of ΔHb and GCS score on hospital mortality was found. TBI patients with a reduced GCS and increased ΔHb have an elevated risk of mortality. The interaction effect was further elucidated by categorzing TBI severity into mild, moderate and severe subtypes based on GCS scores. Compared with that in mild and moderate TBI patients, the mortality risk induced by ΔHb was more pronounced in severe TBI patients, which highlights the importance of maintaining Hb stability in patients with a reduced GCS score.

Fluctuations in Hb during hospitalization are the secondary or combined results of Hb loss, hemoconcentration and hemodilution in TBI patients. Possible explanations for the association between ΔHb and mortality are as follows. Brain becomes vulnerable to changes of hemodynamic in TBI patients with disturbed cerebral autoregulation, impaired cerebral blood flow and pressure (25). Hb decrements may lead to reduced cerebral oxygen saturation and cerebral blood flow (CBF), as well as compromised brain tissue perfusion, thereby exacerbating secondary brain injury (26, 27) and abnormalities of brain tissue respiration (28). Hemoconcentration and hemodilution could result in deteriorating cerebral hemodynamics, contributing to unfavorable TBI outcomes (25, 29, 30). Additionally, high altitude may further weaken the brain's ability to compensate for reduced oxygen delivery via CBF adjustments, making ΔHb-induced harm more difficult to mitigate.

In conclusion, our study confirms the prognostic value of ΔHb, extends this knowledge to TBI patients at high altitude, and identifies a disease- and environment-specific threshold and exponential risk pattern, affected by TBI severity. These findings underscore the need to monitor ΔHb in high-altitude TBI patients and the potential intervention advantages when ΔHb approaches 19.8 g/L, especially in patients with low GCS score, to prevent catastrophic outcomes.

This study has several limitations that should be acknowledged. First, as a single-center retrospective study with limited sample size, potential biases could not be fully eliminated. Second, definitive conclusion could not be drawn due to the observational nature of our study design. The prognostic performance of ΔHb and therapeutic measures to reduce ΔHb with regard to hospital mortality in TBI patients should be investigated in future randomized controlled trials targeting this special population. Third, caution is needed when generalizing our results to high-altitude TBI patients outside Tibetan Plateau region.

## Conclusion

In the context of clinical practice, the findings of our study support that high-altitude TBI patients with a ΔHb > 19.8 g/L, and a significantly elevated ΔHb and a low GCS score have an increased risk of mortality.

## Data Availability

The raw data supporting the conclusions of this article will be made available by the authors, without undue reservation.

## Supplementary material

Supplementary Figure S1 The distribution of ΔHb.
