# Supplementary figures and images for: Association of hemoglobin variation and hospital mortality in patients with traumatic brain injury at high altitude
Source: Front Neurol. 2025 Sep 26;16:1669136. doi: 10.3389/fneur.2025.1669136 (PMC12510844; doi:10.3389/fneur.2025.1669136)

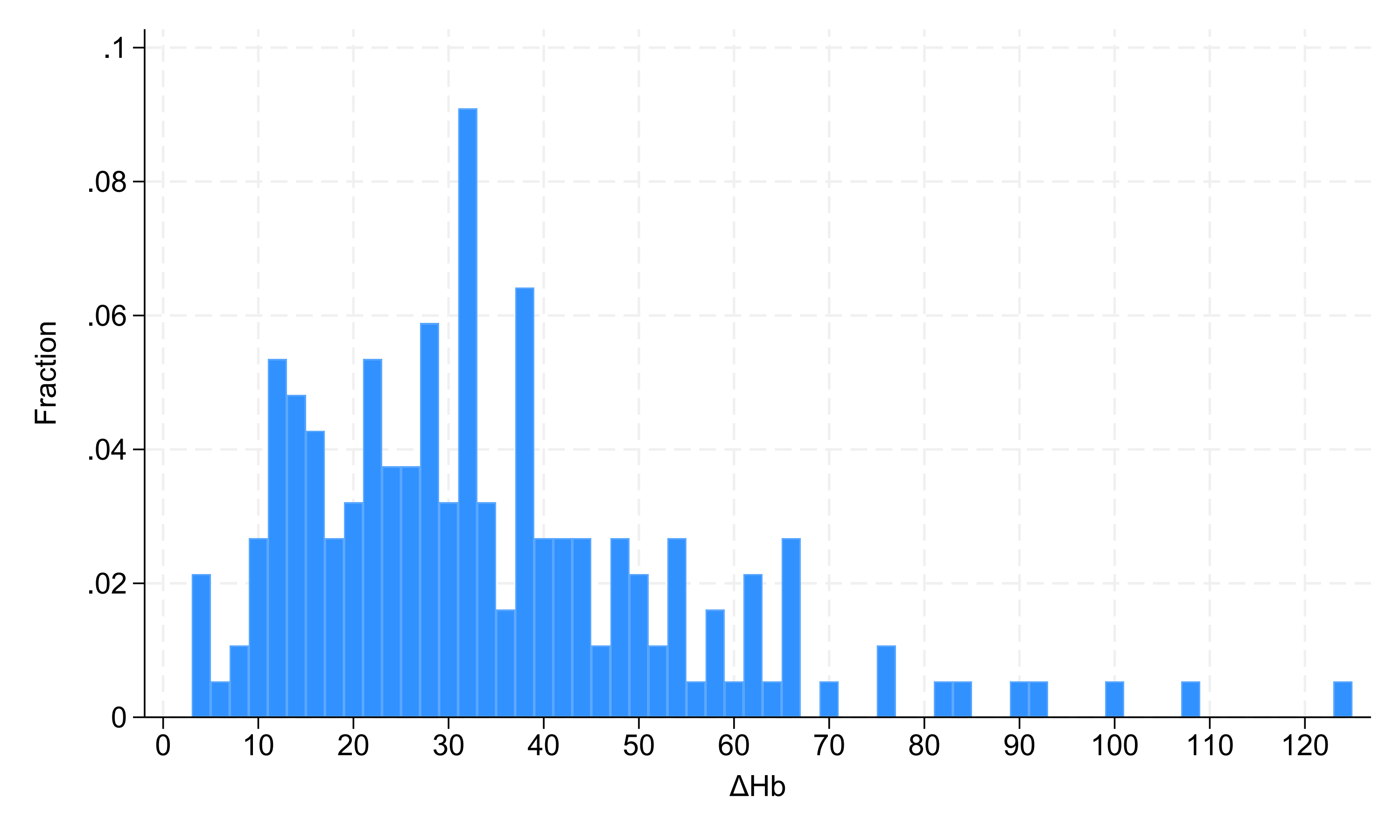

Supplement: Supplementary Figure S1 — The distribution of ΔHb. [file Image_1.tif]
